# Total sleep duration and daytime napping in relation to dementia detection risk: Results from the Million Women Study

**DOI:** 10.1002/alz.13009

**Published:** 2023-04-21

**Authors:** Angel T. Y. Wong, Gillian K. Reeves, Sarah Floud

**Affiliations:** ^1^ Cancer Epidemiology Unit Nuffield Department of Population Health University of Oxford Oxford UK

**Keywords:** daytime napping, dementia, prospective, sleep duration

## Abstract

**Introduction:**

There is inconsistent evidence on the associations of sleep duration and daytime napping with dementia risk.

**Methods:**

In the Million Women Study, a total of 830,716 women (mean age, 60 years) were asked about sleep duration (<7, 7–8, >8 hours) and daytime napping (rarely/never, sometimes, usually) in median year 2001, and were followed for the first hospital record with any mention of dementia. Cox regression estimated dementia detection risk ratios (RRs) during 17‐year follow‐up in 5‐year intervals.

**Results:**

With 34,576 dementia cases, there was strong attenuation over follow‐up in the RRs related to long sleep duration (>8 vs 7–8 hours) and usually napping (vs rarely/never). Short sleep duration was modestly, positively associated with dementia in the long term (RR = 1.08, 95% confidence interval [CI] 1.04–1.12).

**Discussion:**

There was little evidence to suggest that long sleep duration and regular napping are associated with long‐term dementia risk. Short sleep duration was modestly associated with dementia risk, but residual confounding cannot be excluded.

**Highlights:**

- Long sleep duration was not associated with long‐term dementia risk.
- Daytime napping was not associated with long‐term dementia risk.
- There is some evidence for a small higher risk of dementia related to short sleep.

## 1 BACKGROUND

Disturbed sleep is common in patients with dementia.^1^ This may be due to pathological changes in the brain regions controlling circadian rhythms and/or sleep, or to behavioral changes caused by dementia that reduce the synchrony between circadian rhythms and sleep.^2^ However, disturbed sleep is proposed as a risk factor for dementia,^3^ and practicing good sleep hygiene has been suggested as a means for preventing dementia.^4^

Among the various aspects of disturbed sleep, sleep duration was assessed in relation to dementia risk in some cohorts, but the overall evidence is inconsistent. Short sleep has been suggested to increase the risk of dementia potentially through reduced glymphatic clearance of metabolite waste, inflammation, or other mediating mechanisms.^5^ However, a 2019 meta‐analysis of prospective studies found that long, but not short, sleep duration was associated with a higher dementia risk,^5^ whereas another 2019 meta‐analysis of prospective studies, which included cognitive decline as well as dementia as an endpoint, suggested that both short and long sleep were risk factors.^6^ More recently, two prospective studies reported that short, but not long, sleep duration was associated with higher dementia risk, and another two studies (one of which had ≈5000 dementia cases^7^) using data from UK Biobank reported that both short and long sleep were associated with higher dementia risks.^4^, ^7^, ^8^, ^9^ Two recently published studies reported a higher dementia risk associated with actigraphy‐measured daytime napping or self‐reported dozing off during the daytime unintentionally,^9^, ^10^ but a Mendelian randomization analysis reported that daytime napping was associated with a lower Alzheimer's disease risk.^11^

Dementia has a long development period, so early symptoms of disease may cause changes in sleep some years prior to clinical diagnosis, leading to spurious associations between sleep and dementia risk, especially in the short term. In the 2019 meta‐analysis,^5^ only three studies that examined the sleep duration–dementia relationship^12^, ^13^, ^14^ had a follow‐up of 10 or more years, and their overall estimates were weaker compared to those from four combined studies with <10 years of follow‐up, suggesting some degree of reverse causality. Furthermore, dementia is pathologically heterogeneous, so the different types of dementia are likely to have different risk factors. However, only a few published studies have reported on the association between sleep and the risk of Alzheimer's disease,^12^, ^13^, ^14^, ^15^, ^16^, ^17^ with only one study having reported on vascular dementia.^16^

To assess if the associations of sleep characteristics with dementia risk are likely to be due to pre‐clinical disease, as opposed to representing causal risk factors, we examined dementia risk for sleep duration and for daytime napping in relation to follow‐up time in the Million Women Study.

## 2 METHODS

### 2.1 Study

The Million Women Study recruited 1.3 million UK women, 50 to 64 years of age, in median year 1998 (interquartile range [IQR], 1997–1999).^18^ Ethical approval was granted by East of England‐Cambridge South Research Ethics Committee. All women consented to re‐contact and follow‐up via medical records. Information on data access is provided on the Million Women Study website.

**RESEARCH IN CONTEXT**

1. **Systematic Review**: A meta‐analysis of ≈40,000 participants from eight studies reported a positive association with dementia or Alzheimer's disease risk for long versus normal sleep duration. Limited prospective analyses and Mendelian randomization analyses reported conflicting results on the association of daytime napping with dementia risk. We report sleep patterns in relation to dementia risk in ≈800,000 women (≈34,000 cases) over a mean follow‐up of 17 years in the Million Women Study.
2. **Interpretation**: The substantial increases in the short‐term risk of dementia associated with long sleep (vs 7–8 h) and regular napping (vs rarely/never) were attenuated substantially with increasing follow‐up, probably due to reverse causation. Although short sleep duration may be associated with higher dementia risk, its effect is, at most, likely to be very small.
3. **Future Direction**: Further large studies are needed to clarify whether the small association of short sleep duration with dementia risk is causal.

At recruitment, participants completed a written questionnaire including information on sociodemographic, anthropometric, lifestyle, and health factors, and participants were resurveyed at ≈3 to 5 yearly intervals. We used data on sleep characteristics collected in the median year 2001 (IQR, 2000‐2003) (hereafter called the 2001 questionnaire), in which women were asked about their sleep for the first time. The exposures of interest were total sleep duration (based on responses to the question “about how many hours sleep do you get in every 24 hours [please include naps]” [1–23 hours as valid integer values], grouped into <7 hours [short sleep], 7–8 hours [normal sleep, reference group], >8 hours [long sleep]); and daytime napping frequency (based on the question “do you have a nap during the day?” with possible responses grouped as “rarely/never” [the reference group], “sometimes,” and “usually”).

Follow‐up for the disease was carried out via electronic linkage to National Health Service (NHS) databases (≈1% of women have been lost to follow‐up).^19^ The primary endpoint was the first mention of any dementia in a hospital record according to the International Classification of Diseases (ICD)‐10 codes F00‐F03 and G30. For dementia subtypes, the endpoints were the first mention attributed to Alzheimer's disease (ICD‐10 F00 and G30), to vascular dementia (ICD‐10 F01), and to dementia of unspecified type (ICD‐10 F03), respectively. Among women with no hospital record of dementia, 1478 had dementia mentioned in their death certificates, and although they are not included as dementia cases in the main analyses, they were included as cases in sensitivity analyses. A small number of women (242) had both Alzheimer's disease and vascular dementia coded in the first admission for dementia, and so were not coded as cases attributable to either subtype in the subtype analysis. However, the 830 women who had dementia of unspecified type and/or dementia classified elsewhere, together with Alzheimer's disease or vascular dementia, recorded in the first admission, were coded as cases attributable to Alzheimer's disease or vascular dementia, respectively, in the subtype analysis.

Women were excluded from the analyses if they had: any hospital record of dementia (ICD‐9:331.0, 290.0‐290.4, and 294.1; ICD‐10:F00‐F03 and G30) on or prior to the date of questionnaire completion, any missing data on any sleep characteristics, or reported use of sleeping pills over the past 4 weeks, because their baseline sleep pattern may have been altered by sleeping pills.

### 2.2 Statistical analyses

Crude summary statistics were calculated for selected personal characteristics across categories of each sleep characteristic. Using time in study as the underlying time variable, Cox regression was used to estimate hazard ratios (hereafter called dementia detection risk ratios [RRs]) with 95% confidence intervals (CIs) in relation to each sleep characteristic. Women‐years were calculated from the date of completion of the 2001 questionnaire to the earliest of: the first mention of dementia in a hospital record, death, loss to follow‐up, or end of follow‐up (12/31/2019).

Given that in our previous analysis some lifestyle factors appeared to be affected by the pre‐clinical phase of dementia, we adjusted for lifestyle factors recorded on the recruitment questionnaire in the median year 1998,^20^ including deprivation (in fifths, based on the Townsend Index^21^), educational attainment (tertiary qualifications, secondary qualifications, technical qualifications, completed compulsory schooling with no qualifications, or did not complete compulsory schooling), frequency of strenuous exercise (rarely/never, <1, 1–3, >3 times per week), body mass index (BMI) (<20.0, 20.0–24.9, 25.0–29.9, 30.0+ kg/m^2^), smoking status (never, past, current [<10 cigarettes/day], current [10+ cigarettes/day], not current), alcohol consumption (<1, 1 to <3, 3 to <7, 7 to <15, 15+ units/week), and use of menopausal hormones (never, past, current). In addition, some adjustment covariates were available only from the 2001 questionnaire: self‐rated health (poor, fair, good, excellent), which could be a confounder because it is a proxy for morbidities that may change sleep duration; currently married or living with a partner (yes, no); paid work (full‐time, not in paid work, part‐time); and self‐reported treatment for diabetes, high blood pressure, and depression/anxiety (yes/no, for each condition). Women with missing data on a covariate were grouped into an “unknown” category for that covariate (each variable had ≤5% missing data).

The Cox model was routinely stratified by year of birth, year of completion of the 2001 questionnaire, and region at recruitment. Starting from this minimally adjusted model, we assessed the impact of adjustment for sociodemographic and lifestyle factors (educational attainment, deprivation, BMI, strenuous exercise, smoking status, alcohol consumption, use of menopausal hormones, paid work, and currently married or living with a partner), pre‐existing diseases (depression/anxiety, diabetes, high blood pressure), and self‐rated health, by examining the change in the likelihood ratio χ^2^ statistic for the sleep exposure. We estimated the multivariable‐adjusted RRs of sleep characteristics for dementia detection risk (and also for the main types of dementia) during follow‐up intervals of <5, 5–9, 10–14, and 15+ years after baseline.

We conducted the following sensitivity analyses for dementia detection using the multivariable‐adjusted model only in the follow‐up period of 15+ years: (a) complete case analysis in 696,970 women with information on all adjustment variables; (b) analysis with the additional inclusion of death with dementia on the death certificate but not in any hospital record (764 cases occurred during 15+ years of follow‐up); (c) analysis restricted to women who did not report treatment for depression/anxiety, as mental health conditions have been thought to confound the association of interest^4^; and (d) analysis restricted to women who rated their health as good or excellent. For the analysis of total sleep duration, we conducted a sensitivity analysis restricted to women who reported rarely/never napping. As it has been suggested that the association of sleep duration with dementia risk might vary by age,^4^ we also tested for heterogeneity in the main findings between women ≤65 years of age (midlife^3^) and >65 (late life^3^) at baseline.

A follow‐up questionnaire sent in median year 2006 (IQR, 2006‐2006) (hereafter called the 2006 questionnaire) asked women about their sleep (sleep duration: “How many hours each day do you usually spend sleeping? (including at night and naps)” and daytime napping: “Do you have a nap during the day”) as well as self‐rated memory (excellent, good, fair, poor). Self‐rated memory is associated with future cognitive impairment,^22^ possibly reflecting preclinical dementia. It is also possible that cognitive decline may affect how women perceive their sleep, thus affecting the reliability of self‐reported sleep duration. We performed a sensitivity analysis using sleep characteristics collected at both the 2001 and 2006 questionnaires, and restricted to women who (a) returned sleep characteristics (sleep duration within 1–23 hours and daytime napping) in both questionnaires, (b) did not report use of sleeping pills in either questionnaire, (c) rated their memory as good or excellent in the 2006 questionnaire, and (d) reported the same category of sleep duration (i.e., repeatedly short, repeatedly normal, repeatedly long) in both questionnaires. The mean follow‐up was 12.7 years, but in view of possible reverse causation bias we excluded the first 10 years of follow‐up after women reported on their sleep in 2006 and adjusted for the same set of covariates used in the main analysis.

A two‐sided *p*‐value of 0.05 was regarded as statistically significant. Analyses were performed in Stata 17.0 (Stata Corp LP, College Station, TX). Plots were produced using “Jasper makes plots” package (version 2‐266)^23^ in R 4.2.1 (R Foundation for Statistical Computing, Vienna, Austria).

## 3 RESULTS

Of the 866,421 women included in the sample, 88 were excluded based on dementia records in the hospital data prior to baseline, 9919 and 4813 were excluded because of missing data on sleep duration and daytime napping, respectively, and 20,885 were excluded based on reported use of sleep medications, leaving 830,716 women (mean age 60 years [SD 4.9]).

Women most commonly reported normal sleep duration (7–8 hours) (67%), with 23% reporting short sleep duration (<7 hours), and 10% reporting long sleep duration (>8 hours), respectively. The proportions of women reporting usually, sometimes, and rarely/never daytime napping were 6%, 39%, and 55%, respectively.

Women who reported short sleep or long sleep duration (vs 7–8 hours of sleep), and women who reported sometimes/usually napping (versus never/rarely napping) were more likely to be older, have no educational qualifications, have a higher BMI, be a smoker, have ever used menopausal hormones, not be in paid work, and most notably, report poor/fair self‐rated health and current treatment for all selected illnesses (Table 1).

**TABLE 1:** Personal characteristics of 830,716 women and dementia detection during follow‐up, by total sleep duration and by daytime napping.

Personal characteristics, % unless specified otherwise	Total sleep duration			Daytime napping		
	<7 h	7–8 h	>8 h	Rarely/never	Sometimes	Usually
Number of women	190,471	555,500	84,745	455,824	322,849	52,043
Sociodemographic and lifestyle factors						
Age (years), mean (SD)	59.9 (5.0)	59.9 (4.9)	60.8 (5.0)	59.5 (4.8)	60.5 (5.0)	61.5 (5.3)
No educational qualifications	41	35	45	35	40	44
Most deprived fifth	19	15	19	15	18	22
Rarely/never performed strenuous exercise	47	42	52	42	46	54
Current smoker	16	15	17	14	16	19
Body mass index ≥ 30 kg/m^2^	19	15	20	14	19	23
Alcohol consumption ≥15 units per week	5	5	6	6	5	6
Ever menopausal hormone therapy use	53	50	52	49	53	54
Not married or living with a partner	24	18	18	18	20	21
Not in paid work	54	54	71	50	60	70
Health‐related variables						
Treated for depression/anxiety	7	5	10	5	7	11
Treated for diabetes	4	3	5	2	4	7
Treated for high blood pressure	24	21	26	19	25	30
Poor/fair self‐rated health	32	19	31	18	27	43
Follow‐up						
Follow‐up (years), mean (SD)	16.5 (3.5)	16.7 (3.5)	16.0 (4.1)	16.8 (3.3)	16.4 (3.7)	15.6 (4.4)
All dementia cases, % (*n*)	4 (8374)	4 (21,652)	5 (4550)	4 (16,390)	5 (14,899)	6 (3287)
Alzheimer's disease, % (*n*)	1 (2620)	1 (7332)	2 (1415)	1 (5777)	1 (4655)	2 (935)
Vascular dementia, % (*n*)	1 (1437)	1 (3510)	1 (819)	1 (2533)	1 (2644)	1 (589)
Dementia type unspecified, % (*n*)	2 (3922)	2 (9701)	2 (2100)	2 (7259)	2 (6854)	3 (1610)

Over a mean 16.6 years of follow‐up, there were 34,576 cases detected in 830,716 women, with a mean age at diagnosis of 78.3 (SD, 5.6) years (Table 1). During the first 5 years of follow‐up, 798 women had their first hospital admission with mention of dementia; during follow‐up years 5 to 9, a further 4340 did so; during follow‐up years 10 to 14, a further 14,292 did so; and during follow‐up years 15+, a further 15,146 did so.

**FIGURE 1:**
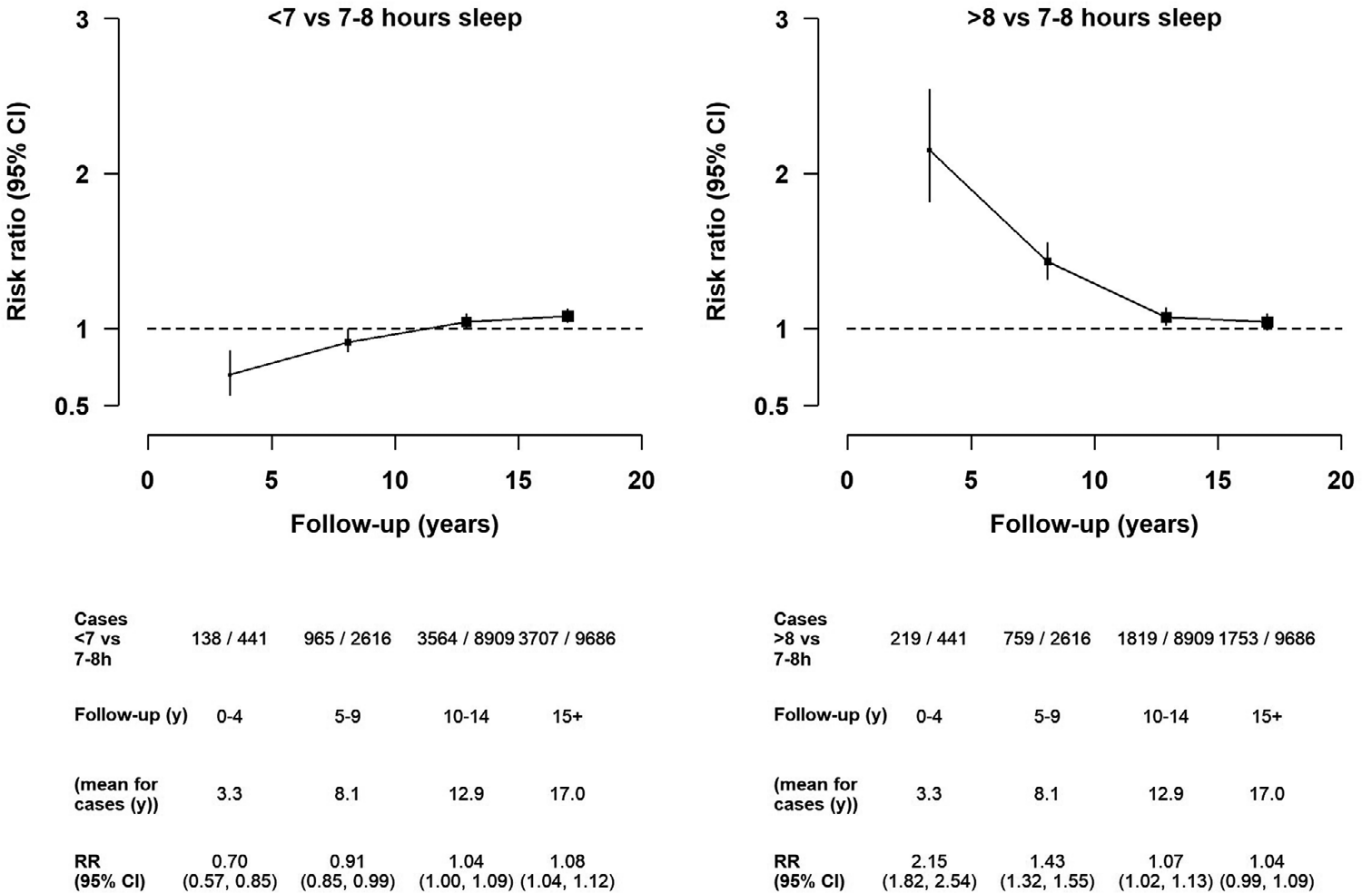
Associations of short and long sleep duration groups with dementia detection risks by period of follow‐up. All multivariable‐adjusted estimates were stratified by year of birth, year of returning the 2001 questionnaire, and region at recruitment, and were adjusted for deprivation, educational attainment, frequency of strenuous exercise, body mass index, smoking status, alcohol consumption, use of menopausal hormones, paid work, currently married or living with partner or alone, depression/anxiety, diabetes, high blood pressure, and self‐rated health. CI, confidence interval; RR, risk ratio; Y, year.

During the first 5 years of follow‐up, short versus normal sleep duration was associated with a lower dementia risk (RR = 0.70 [95% CI, 0.57–0.85]), but this changed to a positive association during 15+ years of follow‐up, with short sleep duration associated with a marginally higher dementia risk (1.08 [1.04–1.12]). Women who reported long versus normal sleep duration had about a 2‐fold higher dementia detection risk (2.15 [1.82–2.54]) in the first 5 years of follow‐up, yet the RR declined substantially to null during 15+ follow‐up years (1.04 [0.99–1.09]) (Figure 1). Due to the notable changes in RRs over follow‐up time, we focused on findings for risks in the period 15+ years after women reported sleep‐related exposures, as these would be least likely to be affected by reverse causality. There was no suggestion of violation of the proportional hazards assumption using Schoenfeld residuals in the multivariable‐adjusted model restricted to 15+ years of follow‐up. Examination of χ^2^ test statistics showed that adjustment for lifestyle and sociodemographic factors weakened the minimally adjusted associations, as did further adjustment for health‐related variables (Table A1).

**FIGURE 2:**
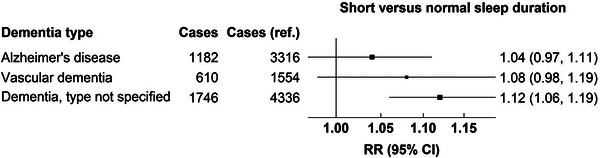
Associations of dementia‐detection risk for short sleep duration during 15+ years of follow‐up by subtype of dementia. CI, confidence interval; RR, risk ratio.

**FIGURE 3:**
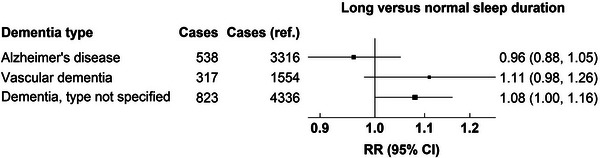
Associations of dementia detection risk for long sleep duration during 15+ years of follow‐up by subtype of dementia. CI, confidence interval; RR, risk ratio.

Of the cases occurring beyond 15 years of follow‐up, 5036 were Alzheimer's disease, 2481 were vascular dementia, and 6905 were dementia of unspecified type. The associations did not appear to differ across dementia types for short versus normal sleep duration (Figure 2) or for long versus normal sleep duration (Figure 3).

In sensitivity analyses restricted to women who reported good/excellent health, and to women without depression/anxiety, the associations were broadly consistent with the main results (Table A1). There was a modest positive association for short sleep with dementia risk in women ≤65 years of age but not in women >65 years of age (*p* for heterogeneity = 0.008) (Table A2).

In the sensitivity analysis based on sleep duration reported at both the 2001 and 2006 questionnaires, 558,824 women with no dementia records when they returned the 2006 questionnaire were eligible for the analysis. Of these women, 154,278, 15,632, and 5030 women were excluded because of poor/fair or unknown self‐rated memory, invalid/missing data on sleep characteristics, and use of sleeping pills, respectively. We further restricted the analysis to 209,615 women who reported the same categories of sleep duration and daytime napping at both questionnaires, among whom 15%, 80%, and 6% reported short, normal, and long sleep duration, respectively. There were 3234 dementia cases in the period 10+ years after completion of the 2006 questionnaire, and the results were consistent with the main findings (RR for short sleep = 1.18 [1.07–1.29]; RR for long sleep = 1.09 [0.96–1.25]).

**FIGURE 4:**
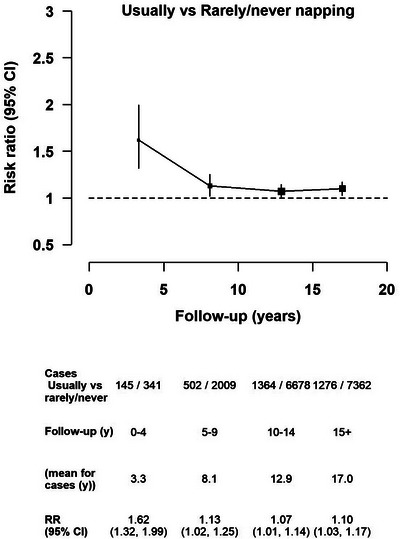
Associations of “usually” versus “rarely/never” daytime napping with dementia‐detection risk by period of follow‐up. All multivariable‐adjusted estimates were stratified by year of birth, year of returning the 2001 questionnaire, and region at recruitment, and were adjusted for deprivation, educational attainment, frequency of strenuous exercise, body mass index, smoking status, alcohol consumption, and use of menopausal hormones, self‐rated health, currently married or living with a partner or alone, paid work, and self‐reported treatment for diabetes, high blood pressure, and depression/anxiety (for each medical condition). CI, confidence interval; RR, risk ratio; Y, year.

For daytime napping, there was no clear association for dementia risk with “sometimes” versus “rarely/never” napping (Figure A1). However, “usually” napping was associated with a 60% higher detection risk compared to “rarely/never” napping in the first 5 years of follow‐up, which then decreased rapidly and remained modest in the subsequent follow‐up periods (RR = 1.10 [1.03–1.17]) (Figure 4). Subsequent analysis focused on risks in the period 15+ years from reporting of napping.

There was notable attenuation of the RR for “usually” versus “rarely/never” napping upon adjustment for sociodemographic and lifestyle factors, and further attenuation upon additional adjustment for pre‐existing illnesses and self‐rated health (Table A3). The test for Schoenfeld residuals did not suggest violation of the proportional hazards assumption in the multivariable‐adjusted results.

**FIGURE 5 alz13009-fig-0005:**
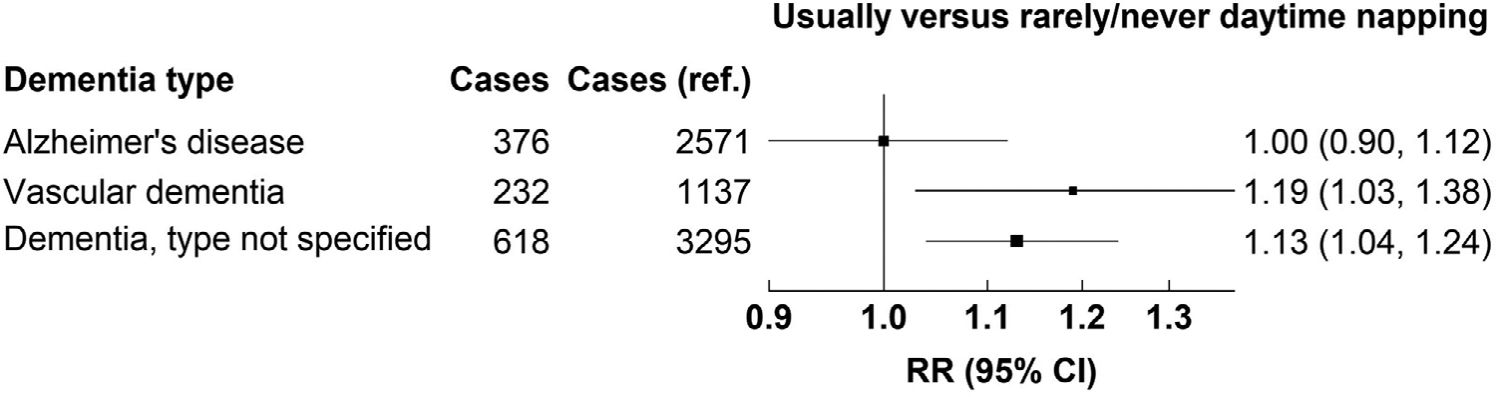
Associations of dementia detection risk for “usually” versus “rarely/never” daytime napping during 15+ years of follow‐up by subtype of dementia. CI, confidence interval; RR, risk ratio.

The sensitivity analyses yielded similar results (Table A3), and there was no evidence of heterogeneity by age group for the overall associations (Table A4). In addition there did not there appear to be heterogeneity by subtype for “usually” versus “rarely/never” napping (Figure 5) or for “sometimes” versus “rarely/never” napping (Figure A2).

Of the 209,615 women who reported the same sleep characteristics in both questionnaires and rated their memory as good/excellent, 67%, 30%, and 3% women reported “rarely/never,” “sometimes,” and “usually” napping, respectively. When the first decade of follow‐up was excluded, the RR (95% CI) for “sometimes” versus “rarely/never” napping was 0.94 (0.87–1.01) and that for “usually” versus “rarely/never” napping was 1.00 (0.84–1.19).

## 4 DISCUSSION

In this large prospective study, more than 0.8 million women reported their sleep characteristics and were followed for an average of 17 years. There were more than 34,000 dementia cases detected, with over 15,000 cases detected after 15 or more years of follow‐up. In the first 5 years of follow‐up there were large positive associations of long versus normal sleep duration, and of usually versus rarely/never napping during the day, with dementia risk, but these were much diminished with longer follow‐up time. Long sleep duration and regular napping may well be consequences of pre‐clinical, or undetected, dementia, because of the insidious and slow onset of dementia. There was some suggestion of a higher dementia risk for short sleep duration, which was stronger in sensitivity analyses aimed at identifying those with more persistent sleeping patterns. However, because the magnitude of the association was small and the association attenuated upon adjustment, residual confounding remains a plausible explanation.

Deprived sleep is hypothesized to[Fig alz13009-fig-0005] increase amyloid beta (Aβ) burden by reducing glymphatic clearance,^24^ and short sleep duration has been found to be associated with poorer cognitive performance and less favorable brain structure measures compared to 6 to 8 hours of sleep.^25^ Four studies,^4^, ^9^, ^14^, ^26^ including one that recorded 521 cases over 25 years of follow‐up,^4^ have reported a positive association between short sleep duration and dementia risk, but two of the studies had fewer than 100 cases in the short sleep groups.^14^, ^26^ In our analysis, there were more than 3000 women who reported short sleep duration after excluding the first 15 years of follow‐up, providing greater statistical power. The inverse associations of short sleep duration with risk in the first 5 years of follow‐up were likely due to reverse causality, as long sleep duration might be a pre‐clinical marker of dementia. The positive associations of short sleep duration with risk of dementia 15 or more years later after minimal adjustment were weakened substantially when all covariates were adjusted for, implying that residual confounding cannot be excluded due to imperfect, self‐reported measurement of important confounders. We found heterogeneity in the association by age at baseline, which has also been reported in the Whitehall II study.^4^ Future studies should consider mechanisms that might explain this heterogeneity by age.

To our knowledge, there is no clear biological hypothesis to link long sleep duration to the development of dementia, although some Mendelian randomization analyses have reported that longer sleep was associated with poorer reaction time and visual memory,^27^ and some analyses have reported non‐linear associations of sleep duration with executive function and brain structure measures,^25^, ^28^ implying that participants with long sleep also had a less‐favorable cognitive profile than participants with normal sleep. Nonetheless, in the current study, the positive association in the short term declined to null as follow‐up time increased. Our findings are in line with those from a previous Swedish study in which an apparent positive association of >9 versus 7.1 to 9 hours in bed per night with dementia risk was no longer significant after restricting to the period 10 or more years after reporting of sleep duration.^13^ In addition, as there was no strong evidence to suggest differences in the associations across dementia types, transitioning to longer sleep might be a non‐specific symptom or early marker of dementia.^12^

Excessive daytime sleepiness is often observed in dementia patients,^29^ and daytime sleepiness is closely related to daytime napping. Our findings add to the limited literature on the relationship between daytime napping and dementia risk by showing results with much longer follow‐up. Two prospective studies, with 3‐ and 10 years of follow‐up, reported a positive association of daytime sleepiness with dementia risk and with vascular dementia risk, respectively, and another prospective study with a median 10 years of follow‐up reported that unintentionally dozing off/falling asleep in the daytime was associated with higher dementia risk.^9^, ^30^, ^31^ Two studies that assessed daytime napping using actigraphic measures reported that long naps of 120+ versus <30 min were associated with cognitive impairment assessed at 12 years^32^ and that a longer nap was associated with a higher dementia risk for up to 14 years of follow‐up,^10^ respectively. Our findings for “usually” napping in the first 5 years of follow‐up were consistent with these publications, but our findings during 5 to 9 years and 10 to 15 years of follow‐up were not. To our knowledge there are no studies with sufficient follow‐up beyond 15 years with which to compare our findings. There was no evidence of material differences in the napping‐associated RRs across subtypes of dementia, and residual confounding could account for the remaining association with vascular dementia risk due to imperfect adjustment for established cardiovascular risk factors such as diabetes and high blood pressure.

The primary strengths of this analysis are the long and virtually complete follow‐up, and the extremely large number of dementia cases included (>34,000), which is more than all cases combined in the latest meta‐analysis.^5^ Although the findings were based on women only, there is no strong evidence for a difference in the association of sleep with dementia risk by sex.^4^ Although we were able to reliably assess the role of sleep duration and napping for dementia risk, other sleep‐related factors that have also been associated with dementia risk, such as sleep quality,^6^ were recorded only 12 years after recruitment, and, therefore, further follow‐up would be required to reliably examine the causal relevance of sleep quality for dementia risk.

## 5 CONCLUSION

Long sleep duration and regular daytime napping were not associated with long‐term risk of dementia. There was weak evidence that short sleep duration may be associated with some increase in dementia risk, but any such effect is likely to be small.

## CONFLICT OF INTEREST STATEMENT

Angel T. Y. Wong, Gillian K. Reeves, and Sarah Floud: No conflict of interest to declare. Author disclosures are available in the supporting information.

## Supporting information

Supplementary Information

Supplementary Information
