# Supplementary material for: Total sleep duration and daytime napping in relation to dementia detection risk: Results from the Million Women Study
Source: Alzheimers Dement. 2023 Apr 21;19(11):4978–86. doi: 10.1002/alz.13009 (PMC10955772; doi:10.1002/alz.13009)
Supplement: Supplementary file 1 — Supplementary Information [file ALZ-19-4978-s001.docx]

Appendix

Table A.1. Effect of adjustment on the association between sleep duration and dementia detection risks after excluding the first 15 years of follow-up

|  | **RR (95% CI) for total sleep duration** | | |  |  |
| --- | --- | --- | --- | --- | --- |
|  | **<7 hours** | **7-8 hours** | **>8 hours** | **LR χ^2^ _(d.f.=2)_** | **Reduction in LR χ^2^** |
| Cases | 3707 | 9686 | 1753 |  |  |
| Stratified by region, year of birth and year of questionnaire completion | 1.18 (1.13 - 1.22) | 1.00 | 1.15 (1.09 - 1.21) | 84 |  |
| Adjusted for sociodemographic and lifestyle factors | 1.14 (1.09 - 1.18) | 1.00 | 1.08 (1.03 - 1.14) | 45 | 46% |
| Further adjusted for pre-existing diseases | 1.12 (1.08 - 1.17) | 1.00 | 1.05 (1.00 - 1.11) | 36 | 58% |
| Further adjusted for self-rated health (Multivariable-adjusted) | 1.08 (1.04 - 1.12) | 1.00 | 1.04 (0.99 - 1.09) | 16 | 80% |
| Excluding women who reported napping |  |  |  |  |  |
| Cases | 1968 | 4833 | 561 |  |  |
| RR (95% CI) | 1.09 (1.03 - 1.15) | 1.00 | 1.02 (0.94 - 1.12) |  |  |
| Excluding women who reported treated for depression/anxiety |  |  |  |  |  |
| Cases | 3372 | 9036 | 1544 |  |  |
| RR (95% CI) | 1.08 (1.04 - 1.13) | 1.00 | 1.04 (0.98 - 1.09) |  |  |
| Among women who reported good or excellent self-rated health |  |  |  |  |  |
| Cases | 2132 | 6817 | 1057 |  |  |
| RR (95% CI) | 1.09 (1.04 - 1.15) | 1.00 | 1.03 (0.96 - 1.10) |  |  |
| Complete-case analysis |  |  |  |  |  |
| Cases | 2939 | 7813 | 1357 |  |  |
| RR (95% CI) | 1.09 (1.04 - 1.13) | 1.00 | 1.02 (0.96 - 1.08) |  |  |
| Including death certificates for ascertainment of dementia |  |  |  |  |  |
| Cases | 3876 | 10188 | 1846 |  |  |
| RR (95% CI) | 1.08 (1.04 - 1.12) | 1.00 | 1.04 (0.99 - 1.10) |  |  |

Sociodemographic and lifestyle factors include deprivation, educational attainment, frequency of strenuous activity, body mass index, smoking status, alcohol consumption, use of menopausal hormone therapy, paid work, and currently married or living with partner. Pre-existing disease variables include treatment for depression/anxiety, diabetes, or high blood pressure.

CI: Confidence interval. RR: Risk ratio.

Table A.2. Subgroup analysis for total sleep duration in relation to dementia detection risk by baseline age

|  |  | **Total sleep duration** |  |
| --- | --- | --- | --- |
| **Age at baseline (years)** | **<7 hours** | **7-8 hours** | **>8 hours** |
| <=65 |  |  |  |
| Cases | 2657 | 6670 | 1235 |
| RR (95% CI) | 1.12 (1.07 - 1.17) | 1.00 | 1.07 (1.01 - 1.14) |
| >65 |  |  |  |
| Cases | 1050 | 3016 | 518 |
| RR (95% CI) | 1.00 (0.93 - 1.07) | 1.00 | 0.96 (0.88 - 1.06) |

P for heterogeneity between age groups for <7 vs 7-8 hours sleep: 0.008

P for heterogeneity between age groups for >8 vs 7-8 hours sleep: 0.06

CI: Confidence interval. RR: Risk ratio.

Table A.3. Effect of adjustment on the association between daytime napping and dementia detection risks after excluding the first 15 years of follow-up

|  | **RR (95% CI) for napping frequency** | | | | |
| --- | --- | --- | --- | --- | --- |
|  | **Rarely/never** | **Sometimes** | **Usually** | **LR χ^2^_(d.f.=2)_** | **Reduction in LR χ^2^** |
| Cases | 7362 | 6508 | 1276 |  |  |
| Minimally stratified by region, year of birth, and year of questionnaire completion | 1.00 | 1.10 (1.06 - 1.14) | 1.30 (1.23 - 1.38) | 82 |  |
| Adjusted for sociodemographic and lifestyle factors | 1.00 | 1.05 (1.02 - 1.09) | 1.21 (1.14 - 1.29) | 39 | 53% |
| Further adjusted for pre-existing diseases | 1.00 | 1.03 (1.00 - 1.07) | 1.16 (1.09 - 1.23) | 22 | 74% |
| Further adjusted for self-rated health (Multivariable-adjusted) | 1.00 | 1.01 (0.98 - 1.05) | 1.10 (1.03 - 1.17) | 9 | 89% |
| Excluding women who reported treating for depression/anxiety |  |  |  |  |  |
| Cases | 6896 | 5942 | 1114 |  |  |
| RR (95% CI) | 1.00 | 1.01 (0.97 - 1.05) | 1.09 (1.02 - 1.16) |  |  |
| Restricted to women who reported good/excellent self-rated health |  |  |  |  |  |
| Cases | 5250 | 4119 | 637 |  |  |
| RR (95% CI) | 1.00 | 1.01 (0.97 - 1.05) | 1.05 (0.97 - 1.14) |  |  |
| Complete-case analysis |  |  |  |  |  |
| Cases | 5917 | 5189 | 1003 |  |  |
| RR (95% CI) | 1.00 | 1.02 (0.98 - 1.06) | 1.09 (1.01 - 1.16) |  |  |
| Including death certificates for ascertainment of dementia |  |  |  |  |  |
| Cases | 7761 | 6830 | 1319 |  |  |
| RR (95% CI) | 1.00 | 1.01 (0.98 - 1.04) | 1.08 (1.01 - 1.14) |  |  |

Sociodemographic and lifestyle factors include deprivation, educational attainment, frequency of strenuous activity, body mass index, smoking status, alcohol consumption, use of menopausal hormone therapy, paid work, and currently married or living with partner. Pre-existing disease variables include treatment for depression/anxiety, diabetes, or high blood pressure.

CI: Confidence interval. RR: Risk ratio.

Table A.4. Association between daytime napping and dementia detection risks after excluding the first 15 years of follow-up, by age at baseline

|  |  | Frequency of daytime napping |  |
| --- | --- | --- | --- |
| Age at baseline (years) | Rarely/never | Sometimes | Usually |
| <=65 |  |  |  |
| Cases | 5290 | 4449 | 823 |
| RR (95% CI) | 1.00 | 1.03 (0.99 - 1.08) | 1.13 (1.05 - 1.22) |
| >65 |  |  |  |
| Cases | 2072 | 2059 | 453 |
| RR (95% CI) | 1.00 | 0.96 (0.91 - 1.03) | 1.03 (0.93 - 1.14) |

P for heterogeneity between age groups for sometimes vs rarely/never: 0.07

P for heterogeneity between age groups for usually vs rarely/never napping: 0.2

CI: Confidence interval. RR: Risk ratio.


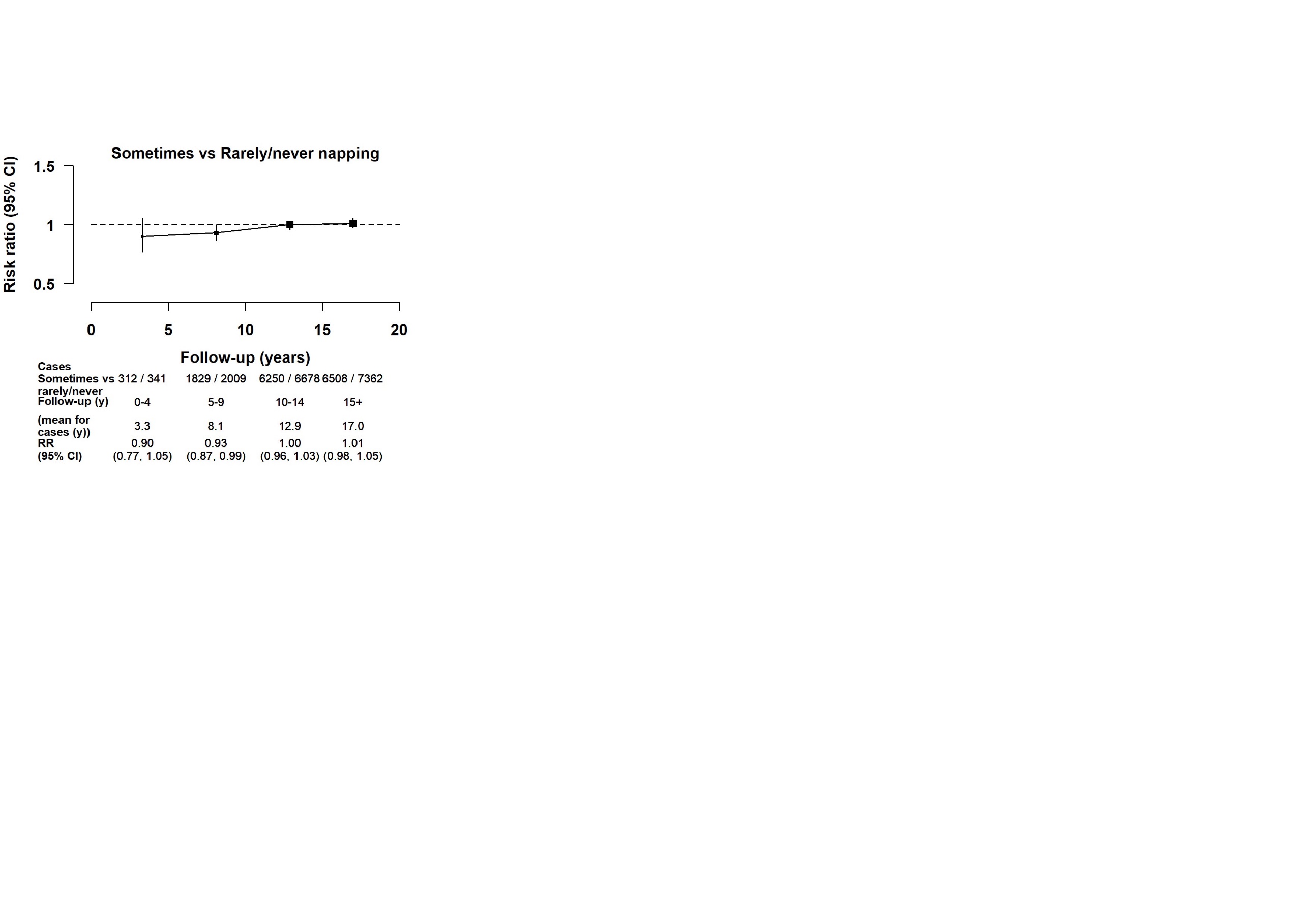


Figure A.1. Association of ‘sometimes’ versus ‘rarely/never’ daytime napping with dementia detection risk by period of follow-up


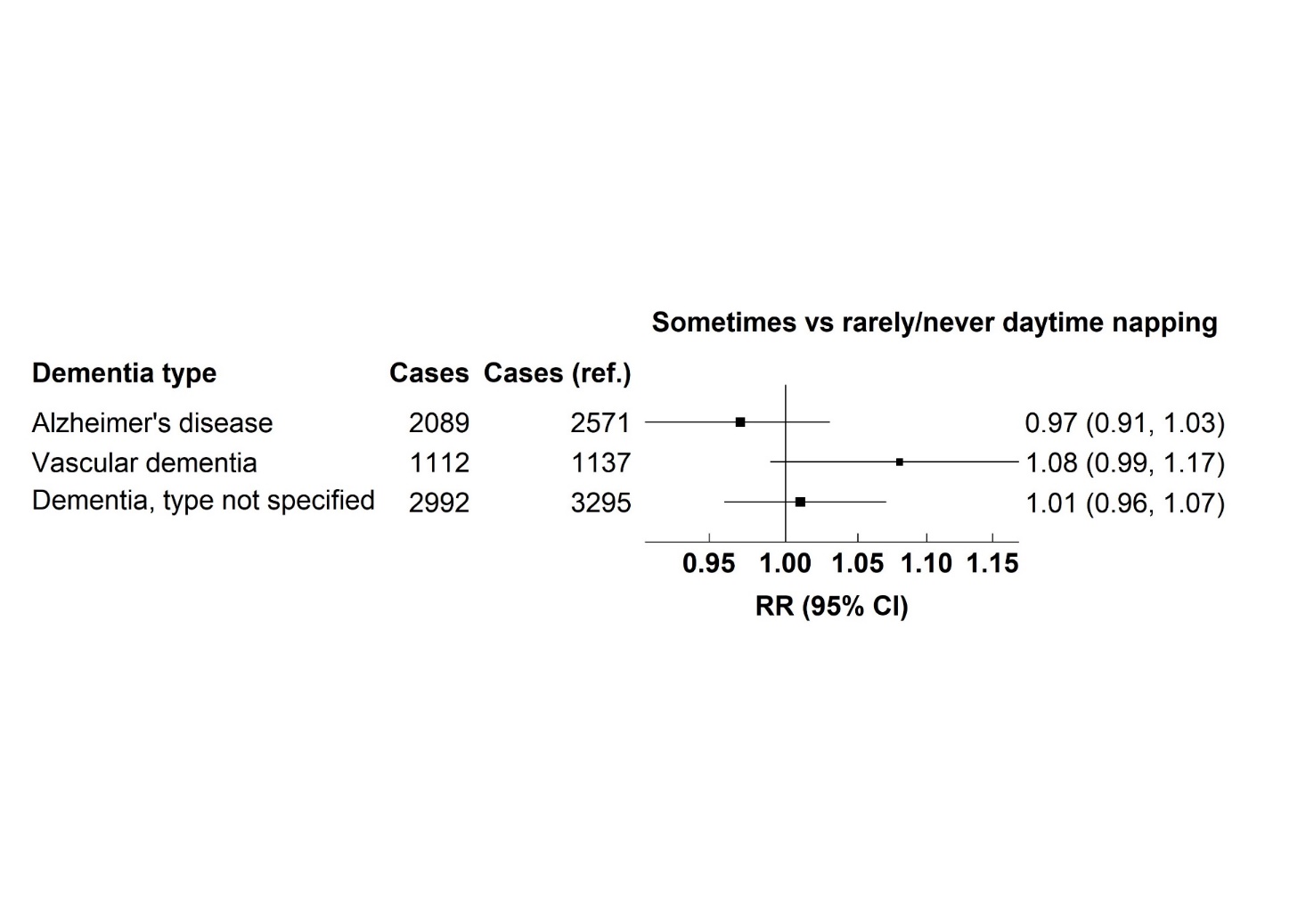


Figure A.2. Associations of dementia detection risk for ‘sometimes’ versus ‘rarely/never’ napping during 15+ years of follow-up, by subtype of dementia
